# Experimental assessment of cross-species transmission in a natural multihost–multivector–multipathogen community

**DOI:** 10.1098/rspb.2023.1900

**Published:** 2023-11-15

**Authors:** Andy Fenton, Susan M. Withenshaw, Godefroy Devevey, Alexandra Morris, Diana Erazo, Amy B. Pedersen

**Affiliations:** ^1^ Institute of Infection, Veterinary and Ecological Sciences, University of Liverpool, Liverpool L69 7ZB, UK; ^2^ Institute of Ecology and Evolution, School of Biological Sciences, University of Edinburgh, Edinburgh EH9 3FL, UK; ^3^ School of Biological Sciences, Cardiff University, Cardiff CF10 3AX, UK; ^4^ Spatial Epidemiology Lab (SpELL), Université Libre de Bruxelles, B-1050 Bruxelles, Belgium

**Keywords:** host community composition, dilution and amplification, perturbation experiment, insecticide treatment, *Bartonella*, *Trypanosoma*

## Abstract

Vector-borne pathogens, many of which cause major suffering worldwide, often circulate in diverse wildlife communities comprising multiple reservoir host and/or vector species. However, the complexities of these systems make it challenging to determine the contributions these different species make to transmission. We experimentally manipulated transmission within a natural multihost–multipathogen–multivector system, by blocking flea-borne pathogen transmission from either of two co-occurring host species (bank voles and wood mice). Through genetic analysis of the resulting infections in the hosts and vectors, we show that both host species likely act together to maintain the overall flea community, but cross-species pathogen transmission is relatively rare—most pathogens were predominantly found in only one host species, and there were few cases where targeted treatment affected pathogens in the other host species. However, we do provide experimental evidence of some reservoir–spillover dynamics whereby reductions of some infections in one host species are achieved by blocking transmission from the other host species. Overall, despite the apparent complexity of such systems, we show there can be ‘covert simplicity’, whereby pathogen transmission is primarily dominated by single host species, potentially facilitating the targeting of key hosts for control, even in diverse ecological communities.

## 1. Introduction

Understanding the dynamics of pathogen transmission within large and diverse wildlife communities is vital for improving surveillance and managing the risk of potential pathogen jumps from wildlife reservoirs into humans or livestock [1–4]. However, given their complexity, we have limited understanding of pathogen transmission dynamics within multispecies ecological communities. This is particularly true for vector-borne pathogens (VBPs), many of which are responsible for some of the most important infectious diseases in humans and livestock (e.g. sleeping sickness, Chagas disease, Lyme disease, etc.), causing significant morbidity, mortality and economic loss. The epidemiology of VBPs is inevitably complex, involving interactions between at least three species: the pathogen, a vector and the definitive host [5,6]. However, the communities in which VBPs circulate often comprise multiple vector and/or host species, magnifying the potential complexity. Within such diverse host and vector communities, there may be both ecological (exposure-related) and physiological (compatibility-related) factors, for both the pathogen and its vectors, influencing the likelihood of transmission in each direction between the vector and host species, and driving patterns of host specialism and generalism across the community [7–11]. In particular, vector species can vary considerably in their propensity to bite different host species, owing to either differential likelihoods of encounters or differences in attraction to the hosts, and different host and vector species can vary considerably in their compatibility with the pathogen. As such, several studies have demonstrated considerable heterogeneities in host species' contributions to VBP transmission, such that a relatively small number of host species may be disproportionately responsible for the maintenance and transmission of a pathogen (e.g. [7,9,12–16]). Hence, the compositions of the host and vector communities, in terms of the abundance, compatibilities and contact routes among them, play a vital role in driving VBP transmission and persistence. Given this, there has been considerable interest, from the perspectives of both applied disease control and the broader field of ecology, in seeking to understand the roles different host and vector species play in driving pathogen transmission and persistence for a range of VBP systems [7–9,12–15,17–21].

To help establish the contributions each host and vector species makes to pathogen transmission, an experimental approach that perturbs transmission pathways can be valuable, potentially allowing the inference of directionality of transmission within and between host species [4,22]. However, experimental perturbations are logistically infeasible for many, if not most, VBP systems, and so have rarely been attempted (but see [23] for an example of vector transmission perturbation in a single-host–single-pathogen system). Here, we present results from a perturbation experiment in a natural multihost–multivector–multipathogen system, focusing on woodland communities of small mammals in northwest England, dominated by two rodent species: wood mice (*Apodemus sylvaticus*) and bank voles (*Myodes glareolus*). Each species is host to a large number of both ecto- and endo-parasites and pathogens [24–28], and they are both abundant in UK woodlands, generally overlapping in distribution, potentially providing opportunity for between-species parasite transmission [29–31]. However, they are not closely related among rodents (likely diverging greater than 20 Ma [32,33]), and exhibit differences in diurnal activity (wood mice being predominantly nocturnal and bank voles crepuscular [30]), microhabitat preference [34,35] and diet (bank voles feeding on fruits and green plants, wood mice more on seeds and invertebrates [36]), potentially reducing opportunities for between-species transmission. Both species are highly amenable to longitudinal sampling [37–42] and manipulation [24] in their natural setting, enabling detailed investigation of the transmission dynamics of their parasites. Here, we focus on their flea-borne pathogens belonging to the genera *Trypanosoma* and *Bartonella*, which are both common in this small mammal community [23,40–43], but are also closely related to species that are pathogenic to both humans and livestock (e.g. *Trypanosoma brucei* causing sleeping sickness in humans and cattle, and *Bartonella henselae* causing cat-scratch fever in humans). We have previously shown that both rodent species at our field sites are hosts to up to seven species of flea, which between them are vectors for two species of *Trypanosoma* [43] and up to five species of *Bartonella* [40–42]. Field observations in this UK system, and related investigations elsewhere, suggest that these pathogens exhibit differing degrees of host specificity to the two rodent species [40–44], and imply that between-species transmission may play varying roles in the persistence of each pathogen (e.g. [41]). However, we know little about vector feeding preferences for the two host species, and so lack information about the roles the vectors play in driving these patterns of host–pathogen association. As such, and consistent with many VBPs, we do not know whether one host species is responsible for driving transmission of these pathogens through the community, or whether observed patterns of pathogen host specificity arise owing to a lack of exposure to, or a lack of compatibility with, the other host species.

To address these issues, we conducted a large-scale field perturbation experiment, using a targeted insecticide treatment applied to either wood mice, bank voles or both species, to disrupt flea, and hence VBP, transmission within and between each host species. Through genetic characterization of host specificity of different pathogen variants, and tracking their responses to our experimental perturbations, we show that the majority of pathogen variants are host specialists, being predominantly found in just one host species, and that there are compatibility barriers that prevent specialist pathogens from infecting other hosts.

## 2. Methods

Here, we present an overview of our methods, leaving additional details to the electronic supplementary material, S1.

### (a) Field methods

Our trapping protocol followed similar methods reported previously [24,39,40]. Wood mice and bank voles were trapped using Sherman live-traps (Alana Ecology, UK; dimensions 8.9 × 7.6 × 22.9 cm) during 2013 and 2014 at two sites in northwest England: Rode Hall (N 53.1213°, E −2.2798°) and The Wirral (N 53.2729°, E −3.0615°). At each site we had four 0.25 hectare trapping grids and each grid had 36 trapping stations, with two traps at each station (72 traps per grid). On all except one grid, traps were set out in a 6 × 6 array with trapping stations placed 10 m apart. Owing to space limitation, trap locations on the remaining grid were set out in an L-shaped formation, but still placed 10 m apart. In both years, trapping took place every three weeks from May to December, resulting in 11 primary (three days within one week) trapping sessions per year (22 trapping sessions in total). For each trapping session, all four grids in one site were trapped in the first week and then all four grids in the other site trapped in the second week. For each session, previously sterilized traps were baited with grain, carrot and bedding and set over three consecutive nights, and all captured animals were identified and processed the following morning.

When first captured, all bank voles and wood mice were given a sub-cutaneous electronic PIT-tag (AVID MicroChips, UK) enabling individual identification. At each capture, standard metrics on each animal were recorded (see electronic supplementary material, S1.1). Individuals were checked visually for ectoparasites by combing the fur over a water bath, recording the number of fleas, ticks and mites, and collecting them. We note that this method is not exhaustive in the field, and some ectoparasites may be missed; hence the data presented likely underestimate their true infestation loads and prevalences. A small blood sample (approx. 25 µl) was taken from the tail tip of each individual at each trapping session (one sample per three weeks) to determine if it was infected with *Bartonella* or *Trypanosoma* species (see below and electronic supplementary material, S1.2).

### (b) Transmission-blocking treatment experiment

In order to determine the within- and between-species transmission dynamics of the fleas and VBPs in this rodent community, we used a grid-level insecticide treatment regime to reduce the flea infestation rates and flea population size on specific grids. We had four treatment types applied at the grid-level, and this design was replicated at each site and across two years (2013 and 2014): (i) ‘mouse-only’ treatment (all mice captured within every session were treated), (ii) ‘vole-only’ treatment (all bank voles captured within every session were treated), (iii) a combined mouse- and vole-treatment grid (50 : 50 treatment), where every other individual captured within a session was treated regardless of species identity, such that approximately 50% of all individuals on the grid in a given session were treated (remaining animals were designated as control animals, receiving water) and (iv) a ‘control’ grid, where no individuals received treatment. Grids retained the same treatment regime across both years.

Individuals that were treated were given a dermal weight-specific dose (10 mg kg^−1^) of the broad-spectrum veterinary insecticide fipronil (Frontline Plus) [45] at their first capture, and then again at each three-weekly trapping session, with the aim of removing their fleas. The liquid treatment was applied topically underneath the chin using a pipette to reduce the possibility that it would be removed by grooming. All untreated animals (whether on a treatment or control grid) received a sham treatment of water applied in the same way. Fipronil is mildly toxic to small mammals (oral LD50 in mice = 95 mg kg^−1^ and dermal LD50 in rats greater than 2000 mg kg^−1^; [46]), but a previous study found no effects of fipronil treatment on the survival of wild field voles, *Microtus agrestis* [23]. Fipronil exerts largely insect-specific neurotoxic effects [47] and kills both adult and larval fleas within 24 h of application [48].

### (c) Identifying fleas and pathogens

#### (i) Flea identification and processing for pathogen identification

All collected fleas were identified morphologically to species using methods described previously [40]. Fleas were stored in 70% ethanol at −20°C until further processing. DNA was extracted from individual fleas using a Promega Wizard Genomic DNA purification Kit (Promega Corporation, USA). One microlitre of each extract was used in a PCR targeting the conserved invertebrate 18S rRNA gene (primers ‘1’ and ‘6’ of Hendriks *et al.* [49]) to confirm DNA extraction success.

#### (ii) Rodent blood processing for pathogen identification

*Trypanosoma* spp. and *Bartonella* spp. infections for all bank voles and wood mice were identified using nested PCR diagnostics from the blood samples taken from each individual at each trapping session. All blood samples collected in the field were returned to the laboratory and spun down in a centrifuge at 12 000 r.p.m. (16 000*g*) for 10 min, and the serum and blood pellet were separated and stored at −20°C until further processing. Details of blood DNA extraction and pathogen identification methods are presented in electronic supplementary material, S1.2.

#### (iii) *Bartonella* classification

We previously identified a high degree of diversity at the pITS region of *Bartonella* infecting rodents at these field sites, and crucially, pITS variants of the same *Bartonella* species were largely host-specific [40]. For this reason, all pITS amplicons were subsequently sequenced to identify the pITS variants of positive samples (variants distinguished by a difference in one or more base pairs), following the methods of Withenshaw *et al*. [40]. We note though that this method is unable to distinguish pITS variants when individuals are co-infected with multiple *Bartonella* variants, which potentially constrains inferences about the full occurrence and diversity of variants in this system.

Owing to the low prevalences of many of the individual *Bartonella* variants, we did not seek to analyse each one separately, but rather grouped them into three functional ‘variant groups’ according to their observed levels of occurrence in the two host species: ‘mouse-specific’ (predominantly found only in wood mice; see below), ‘vole-specific’ (predominantly found only in bank voles) or ‘host-shared’ (broadly found in both host species). When classifying variants into host-specific or host-shared, we note that some variants were found in both host species but had a highly heterogeneous distribution across the host species (i.e. a large majority of samples of that variant were found in just one host species). While any variant that is found in both species could strictly be classified as ‘host-shared’, to do so would group together variants with very different patterns of host occurrence (some variants were highly dominated by wood mouse infections, some highly dominated by bank vole infections). Reasonably, we might assume that if the proportion of all samples for a given variant in one host species is very low, those likely represent rare ‘spillover’ infections, and that variant is predominantly maintained by the other host species. We, therefore, classified a variant as ‘host-specific’ if greater than 95% of the total number of samples for that variant were found in one host species. This classification allows for some (less than or equal to 5%) occurrences in the other host species, assuming those rare occurrences represent spillover infections. If both host species had greater than 5% of the total number of samples for a given variant then it was deemed to be ‘host-shared’. We assessed sensitivity to this 5% threshold by re-running our analyses using a 10% threshold, and found no changes to our inferences about treatment effects. We note that assuming a strict 0% threshold (a variant with any occurrence in both host species is deemed ‘host-shared’) not only combines a highly diverse range of host sharing into a single group, but also results in too few ‘host-specific’ samples for models to converge. Conversely, raising the threshold beyond 10% results in only two variants being deemed ‘host-shared’, preventing those models from converging. We, therefore, retain the 5% threshold for our analyses, as a pragmatic value which acknowledges that different variants found in both host species can have widely divergent patterns of host sharing, while providing sufficient balance of host-shared and host-specific variants to allow all models to converge.

Finally, 12 apparently host-specific variants were very rare, each being found fewer than 10 times in total. It is, therefore, possible that if more samples had been collected for these variants, we may have found positive infections in both host species. We note that the rarest variant that was found in both species had 10 samples (*B. grahamii*-4; electronic supplementary material, table S3). To ensure that uncertainty in classification of these rare (*N* < 10) variants was not biasing our analyses we, first, analysed the data classifying the rare variants as host-specific and then, secondly, re-ran all analyses using an alternative scheme that re-classified those rare variants as being host-shared.

### (d) Statistical analyses

We analysed the data from our experiment using generalized linear mixed models (GLMM), with the following binomial response variables (with logit link): (i) flea infestation status (all flea species pooled; 1 = one or more fleas present; 0 = no fleas present), (ii) *Trypanosoma* infection status (1 = infected; 0 = not infected), (iii) host-specific *Bartonella* infection status (classification scheme described above; see electronic supplementary material, table S3 for classification of variants as host-specific or host-shared) or (iv) host-shared *Bartonella* infection status. For each model we used data only from individuals that had not been previously treated with fipronil (i.e. all individuals on control grids, all individuals of non-target host species on treatment grids, and first (pre-treatment) captures of individuals of target host species on treatment grids). Effectively these untreated animals act as ‘sentinels’ to determine how the grid-level force of infection for fleas or VBPs was affected by the differential targeting of treatment through a four-level ‘Treatment’ variable: (i) mouse treatment, (ii) vole treatment, (iii) 50 : 50 treatment, or (iv) no treatment (control). Since all animals were untreated at session 1, there would be no effect of treatment, so we only used data from trapping session 2 onwards.

For host-specific pathogens (trypanosomes and some *Bartonella* variants, see below), we ran separate analyses for each host species. For host-shared pathogens (fleas and some *Bartonella* variants, see below) we ran models for both host species combined, with a Treatment ∗ Species interaction term, as the effects of the different treatments may differ between mice and voles. To control for non-independence of animals caught at broadly the same place and the same time of year, we included a composite 12-level random effect term, comprising (i) woodland site (Wirral or Rode Hall), (ii) year (2013 or 2014) and (iii) time of year (early (summer) = sessions 1–4; mid (late summer/early autumn) = sessions 5–8; late (late autumn/early winter) = sessions 9–11). For each analysis we also controlled for a number of host demographic characteristics that might influence an individual's infestation or infection risk: (i) age (young (juvenile and sub-adult), adult), (ii) sex (male, female), (iii) reproductive status (active, not active). We also included a two-way interaction between sex and reproductive condition, as reproductive status may affect behaviour/physiology differently for males and females. We explored the effects of including host body condition (a continuous variable, 2–10 from poor to excellent condition) and, for models of pathogen infection risk, flea presence at the time of capture (a categorical variable with two levels, present or absent); however, preliminary analyses showed that including either of these terms had no effect on any treatment effects, and in some cases prevented model convergence. We, therefore, did not include them in the final analyses presented here. All GLMMs were run using the ‘*glmer*’ function from the lme4 package in R (v.4.1.1) [50], with models fitted by maximum likelihood (Laplace approximation).

For host-specific pathogens, we assessed the significance of grid-level treatments on probabilities of infestation/infection directly from the GLMM tables using the control grids as the baseline reference. For host-shared pathogens/fleas, where the model included a Treatment ∗ Species interaction term, we carried out *post hoc* tests to compare for each host species the effect sizes of treated grids in comparison with the corresponding control grids (using Dunnett's adjustment for multiple testing, specified by the ‘trt.vs.ctrl’ option of the ‘*contrasts*’ function within the emmeans R package [51]). All inferences were made based on the above-specified full models; no model simplification or selection was carried out.

We assessed the robustness of our results in several ways. First, to control for possible pseudoreplication due to repeat captures for a subset of animals, we attempted to run generalized linear mixed models, with each animal's ID number as a random effect; however, the models failed to converge. We, therefore, ran the GLMMs described above using a subset of the full dataset that consisted of a single, random capture from each individual. The results of this did not substantially differ from analyses using the full dataset; in particular, the same treatment effects were observed for both the analysis of single-capture data and that of the full dataset. Previous analyses of data from this system have also found little influence of this potential source of pseudoreplication, likely owing to the relatively low number of repeat captures of individuals [52], so we present the results of the full data analysis for simplicity. Furthermore, as mentioned previously, 12 *Bartonella* variants that were designated as host-specific under our original classification scheme (more than 95% of samples found in only one host species) comprised fewer than 10 samples, which may be too small a sample size to detect them in both host species. We, therefore, re-ran all analyses re-classifying those rare variants as ‘host-shared’, but this did not affect our conclusions (see below). Finally, to account for possible seasonal variation in the effect of treatment, we investigated an alternative to the above modelling approach, removing ‘time of year’ from the random effect term, and including it either as fixed main effect or as an interaction term with Treatment. Overall though we found little support for time-dependent treatment effects (see electronic supplementary material, S2.4 for details).

## 3. Results

### (a) Characterization of host-specificity

Data from 1581 wood mice and 1007 bank voles (see electronic supplementary material, S2 and table S1A for details) showed both flea and *Trypanosoma* prevalence to be consistently higher in bank voles (fleas: 16%; *Trypanosoma* spp.: 25%) than wood mice (fleas: 6%; *Trypanosoma* spp.: 7%; electronic supplementary material, table S2). Overall *Bartonella* prevalence was similar in both host species (approx. 50%), although it varied across the different *Bartonella* species (electronic supplementary material, table S2).

**Figure 1.**
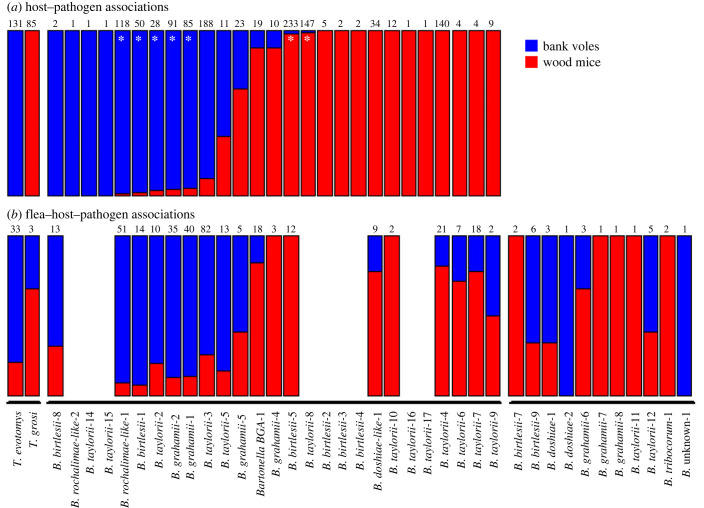
Relative degrees of host specialization for each pathogen (*Trypanosoma* or *Bartonella*) species or variant. The proportions of red (bank voles) and blue (wood mice) in each bar represent the proportion of samples of that pathogen that were either (*a*) found infecting the relevant host species, or (*b*) found in fleas collected from that host species. The numbers at the top of each bar represent the total number of positive host blood (*a*) or flea (*b*) samples for that pathogen. The pathogens are grouped into three categories (denoted by the horizontal lines at the bottom of (*b*)): (i) *Trypanosoma* spp., (ii) *Bartonella* variants found in mice or voles (the corresponding gaps in (*b*) represent variants found in hosts but not in our flea samples), and (iii) variants found in fleas but not in mice or voles. *Bartonella* variants where greater than 95% of samples were found in one host species (marked with ‘*’) were classified as being host-specific to that species; all other variants found in both host species were classified as host-shared (generalists). Host-specific variants with fewer than 10 total samples were subsequently classified as being (potential) host-shared variants in an alternative classification scheme to assess robustness of analyses to potential misclassification of these rare variants.

Four of the six *Bartonella* species appeared to be host generalists, with positive samples collected from both wood mice and bank voles (figure 1; electronic supplementary material, table S3). However, sequencing these samples revealed a higher degree of host-specificity than observed at the pathogen species level. Consistent with our previous findings [40], and other studies in related systems [44], many variants within each of *B. taylorii*, *B. grahamii* and *B. birtlesii* showed complete host specificity for either bank voles or wood mice (figure 1; electronic supplementary material, table S3). We also found complete host specificity of *Trypanosoma* species (electronic supplementary material, table S5); all 131 samples sequenced from bank voles were identical to the published sequence of *T. evotomys* (GenBank accession number AY043356), whereas all 85 sequences from wood mice were identical to *T. grosi* (GenBank accession number AY043355).

In terms of the vectors, we found six flea species, with all but one species occurring on both wood mice and bank voles (the exception was *Palaeopsylla soricis*, which only had one sample, from a wood mouse; electronic supplementary material, table S4). The majority of pathogen species and variants found in the rodents were also found in the fleas (compare figure 1*a*,*b*), although there were some ‘missing’ variants from the flea population. Generally, these missing variants were very rare within the rodent host community (typically one or two samples), although *Bartonella* variant *B. taylorii-8* was the exception, occurring in 147 rodent samples, mostly from wood mice, but never found in any of the fleas sampled. In addition, there were several *Bartonella* variants that were only found in fleas, but not in rodent hosts (figure 1*b*, right-hand group of variants), although again these missing variants from the rodent samples were generally very rare among the fleas. For those variants found in both the fleas and rodents, the patterns of host sharing seen in fleas broadly mirrored that of the variants in the hosts (compare figure 1*a*,*b*, and see electronic supplementary material, figure S1). However, as previously reported [40], there were instances of fleas carrying host-specific pathogens being found on the ‘wrong’ host in this system (i.e. variants only found infecting bank voles were found in some fleas feeding on wood mice, and vice versa). In particular, among the host-specific *Trypanosoma* species, out of 43 fleas that tested positive for *T. evotomys* (the bank vole-specific species), seven were found on wood mice, and out of three fleas that tested positive for *T. grosi* (the wood mouse-specific species), one was found on a bank vole. Hence, the observed patterns of pathogen host specificity are not purely due to lack of exposure to the other host; hosts of one species can be exposed to fleas that have clearly fed on the other host species (enabling them to be infected by the host-specific pathogen from that species), but the host-specific pathogens within those fleas were never found to infect the alternative host in this study.

### (b) Field experiment results

**Figure 2.**
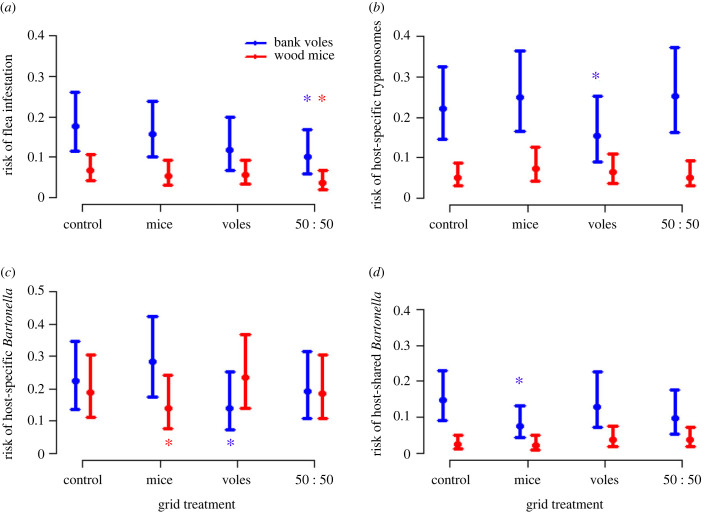
Binomial GLM fitted values (±1.96 × standard error) of grid-level treatments (none (control), mice-only treatment, vole-only treatment, or ‘50 : 50’ (every other animal treated regardless of species identity)) on the risk of (*a*) flea infestation, (*b*) *Trypanosoma* spp. infection, (*c*) host-specific *Bartonella* pITS variant infection, and (*d*) host-shared *Bartonella* pITS variant infection (variants categorized as shown in electronic supplementary material, table S3), in untreated bank voles (blue) and wood mice (red). Asterisks show responses that are significantly different (*p* < 0.05) from animals on the control (untreated) grids in each case.

Based on the minimum number known alive of each species, we caught (and therefore treated, as appropriate) approximately 90% of animals available on each grid in each session (electronic supplementary material, S2.1 and figure S2), suggesting high treatment coverage of our target treatment groups throughout the experiment (although we do recognize that there is high turnover in these populations, with many animals being caught only once, potentially diluting overall treatment effects on our trapping grids; electronic supplementary material, S2.1). The total numbers of each species treated on each grid type are given in electronic supplementary material, table S1B. We found significant effects of grid-level treatment (‘mouse-only’, ‘vole-only’, or combined ‘50 : 50’ mouse and vole treatment) on various measures of pathogen infection and flea infestation risk in untreated ‘sentinel’ animals caught on those grids (electronic supplementary material, tables S6 and S7; figure 2). Interestingly, we found no direct effects of targeted treatment on flea infestation levels in either bank voles or wood mice (i.e. infestation levels on vole-treatment or mouse-treatment grids did not differ from levels on the control grids; electronic supplementary material, table S7; figure 2*a*). However, we did find approximately 45–50% reductions in flea infestation levels for both bank voles and wood mice on the ‘50 : 50’ treatment grids compared with control grids (bank voles: 50 : 50-treatment coefficient = −0.65 ± 0.22, *z* = −2.98, *p* = 0.008; wood mice: 50 : 50-treatment coefficient = −0.62 ± 0.25, *z* = −2.45, *p* = 0.04; electronic supplementary material, table S7; figure 2*a*). Together these results suggest that both host species contribute to maintaining the overall flea community, with neither species playing a significant role on its own.

In terms of the pathogens, significant treatment effects were predominantly ‘target-specific’, being seen primarily on infections of the treated host species (figure 2). In particular, untreated bank voles on vole-treatment grids were approximately 35–45% less likely than voles on control grids to have vole-specific *T. evotomys* infection (vole-treatment coefficient = −0.45 ± 0.23, *z* = −1.97, *p* = 0.048; electronic supplementary material, table S6; figure 2*b*), and vole-specific *Bartonella* variants (vole-treatment coefficient = −0.56 ± 0.25, *z* = −2.22, *p* = 0.026; electronic supplementary material, table S6; figure 2*c*). Similarly, untreated wood mice on mouse-treatment grids were approximately 32% less likely than mice on control grids to be infected with mouse-specific *Bartonella* variants (mouse-treatment coefficient = −0.39 ± 0.20, *z* = −1.96, *p* = 0.05; electronic supplementary material, table S6; figure 2*c*). We emphasize that these reductions in infestation risk were seen in untreated animals on the treatment grids, and so reflect grid-level reductions in the force of infection of fleas and those host-specific pathogens due to treatment of neighbouring animals, which reduce infection risk for untreated animals, rather than the direct effect of treatment on treated animals.

Generally there was little evidence of non-target treatment effects on infection or infestation risks for the untreated host species; wood mice on vole-treated grids and bank voles on mouse-treated grids did not differ in their flea infestation or host-specific pathogen infection risks compared with those animals on control grids (electronic supplementary material, tables S6 and S7; figure 2). However, there was one exception: bank voles on mouse-treatment grids were approximately 50% less likely to have host-shared *Bartonella* variants (mouse-treatment coefficient = −0.75 ± 0.24, *z* = −3.18, *p* = 0.004; electronic supplementary material, table S7; figure 2*d*) compared with bank voles on control grids. It should be noted, however, that we otherwise found little effect of mouse-only treatment overall. There was no detectable effect of mouse treatment on their risk of flea infestation, or on infection with (mouse-specific) *T. grosi* or host-shared *Bartonella* variants (electronic supplementary material, tables S6 and S7; figure 2*a*,*b*,*d*). These pathogens all had very low baseline prevalences of infestation/infection in wood mice (typically less than 10%), and hence there may be low power to detect those treatment effects. Thus the role of wood mice in driving the grid-level force of infection of these species and variants remains somewhat unclear.

Finally, we note that the above findings were not sensitive to our assumptions about classification of *Bartonella* variants, whether re-classifying rare variants (those with fewer than 10 samples) as being host-shared (i.e. considering the possibility that, if more samples had been available for those rare variants, they may have been found in both host species; electronic supplementary material, figure S4), or assuming a 5% threshold delineating between host-specific and host-shared variants (increasing this threshold to 10% did not alter inferred treatment effects; electronic supplementary material, figure S5). Furthermore, as mentioned above, we found little evidence of time-dependent treatment effects (electronic supplementary material, S2.4). Hence, we have some confidence that our conclusions concerning the effects of the different treatment regimes are largely robust to specific assumptions about variant specificity and potential within-year variation in treatment effects.

## 4. Discussion

Our results suggest that pathogen infection risks in this system are determined primarily by intra- rather than interspecific processes within the rodent host community. With some exceptions (detailed below), both bank voles and wood mice appear to largely maintain their own host-specific pathogens (as evidenced by untreated voles on vole-treated grids having a lower risk of host-specific *Trypanosoma* and *Bartonella* infections compared with voles on control grids, and untreated wood mice on mouse-treated grids having a lower risk of host-specific *Bartonella* infections compared with mice on control grids). In all these cases, treatment of a focal host species reduced the grid-level force of infection of those fleas, and hence of the pathogens they carry, sufficiently to reduce infection risk for untreated conspecifics living on those grids. Evidence for cross-species transmission was sparse, although there was a suggestion that both host species play a role in maintaining the overall flea community, and that wood mice play a role in maintaining host-shared *Bartonella* infections in bank voles. Overall, despite the potential complexity of multihost–multivector systems like this, there was broad evidence of ‘covert simplicity’, whereby transmission for most pathogens in the system is primarily dominated by a single host species, potentially facilitating the targeting of key hosts for transmission control.

While intraspecific effects seemed to dominate, there was some evidence for interspecific effects, primarily from wood mice to bank voles. We found a significant reduction in the prevalence of host-shared *Bartonella* variants in bank voles living on mouse-treated grids, suggesting that wood mice may act as a reservoir host for these variants, with bank voles largely being spillover hosts [2–4]. It is notable that these host-shared *Bartonella* variants are predominantly vole-biased (251 samples in total, 186 (approx. 75%) of which were found in voles); hence it is not obvious why mouse-targeted treatment would reduce infection levels of these variants. One possibility is that there are differences between the host species in their propensity to acquire infection from, and their propensity to pass infection to, biting fleas. Hence, numbers of infected animals of each species may not translate into contributions to overall transmission [16]. Furthermore, the flea vectors in systems like this are often nest-dwellers, feeding on whichever hosts enter those nests [53,54]; hence cross-species feeding generally occurs via sequential nest use by hosts. If there is asymmetry in this, for example if bank voles are more likely to use a previous wood mouse nest than wood mice are to use previous bank vole nests, then cross-species transmission would more likely occur from wood mice to bank voles, rather than *vice versa*. Clearly, further work is needed to understand these mechanisms of cross-species transmission, but general theory suggests that for pathogens which show strong reservoir–spillover dynamics, the greatest reduction in infection risk for the spillover host is achieved by targeting the reservoir host (wood mice, in this case), rather than the spillover host (bank voles) itself [1,2]. The results presented here provide a rare experimental demonstration of that effect in a natural multihost community.

In order for wood mice to play a role in maintaining infections of host-shared variants in bank voles, there must be some degree of cross-species feeding by the fleas. As mentioned previously, although wood mice and bank voles are generally sympatric, there are differences in their ecologies, diets and behaviours that may limit opportunities for cross-species flea sharing [30,34–36]. However, as mentioned above, cross-species feeding in systems like this generally occurs via sequential nest use by hosts [53,54], even if direct encounters between host individuals are rare. At a broad taxonomic scale, six of the seven flea species in this system are known to be host generalists, with individuals of all six species being found on both host species [40]. Furthermore, we found that that the ‘50 : 50’ treatment was effective at reducing flea infestation levels of both host species, whereas targeted treatment of either species had no detectable effect (figure 2*a*). As such these results suggest that the two host species largely share a common pool of fleas, with both species playing a role in maintaining the overall flea community. However, this does not reveal finer-scale details about the extent to which individual fleas move between host species; for cross-species pathogen transmission to occur, an individual flea must first bite an infected individual of one host species, and then bite a susceptible individual of the other species. The possibility of this occurring is supported by the finding of host-specific *Trypanosoma* species and *Bartonella* variants (also reported in [40]) inside fleas feeding on the ‘wrong’ host (i.e. a mouse-specific pathogen being carried by a flea feeding on a vole, and *vice versa*). Hence, some degree of cross-species feeding by individual fleas clearly does occur, but then VBP–host incompatibilities likely prevent successful infection of those pathogens in the alternative host.

It is notable that the only detectable reductions in overall flea infestation rates were seen on the 50 : 50 treatment grids, whereas this treatment did not result in any detectable reduction in infection risk of any of the pathogens examined. Conversely, those pathogens where a reduction in infection risk was observed did not occur on treatment grids with an associated detectable reduction in the flea populations. In particular, we observed clear reductions in the prevalences of three of the four host-specific pathogen groups (vole-specific trypanosomes, and vole- and mouse-specific *Bartonella* variants), due to targeted host treatment (mouse-only or vole-only), despite no detectable reduction in flea prevalence on any of those grids. These findings suggest that treating to reduce the overall vector population may not translate to reductions in VBP infection risk. Instead, for such host-specific pathogens, it is more important to target the subset of vectors that have a propensity to bite the relevant host species, thereby reducing the pathogen's force-of-infection on those vectors.

Despite the effects described above, the wider role that wood mice play in the epidemiology of fleas, or the pathogens that they are infected with, is still unclear. Perhaps surprisingly, there was no evidence of mouse treatment on mouse-specific *T. grosi* infections in mice, although there was an effect of mouse treatment on host-specific *Bartonella* variants in wood mice. These inconclusive effects of mouse treatment may be due to a lack of power to detect a difference in host-specific *Trypanosoma* infections, because their prevalences were so low (typically less than 10%). Furthermore, although we saw an effect of mouse treatment on ‘host-shared’ *Bartonella* variants in bank voles, there was no detectable effect on the same variants in wood mice. Again, this could relate to a lack of power as positive samples of these variants in wood mice were quite rare (figure 2*d*), or potential subtleties in the effects of treatment on the different variants within the ‘host-shared’ category, due to the range of host-sharing patterns between them (figure 1; electronic supplementary material, table S3). Other possible explanations for the general lack of effects of mouse treatment may be that the treatment coverage of wood mice may have been insufficient to have a detectable effect (although treatment coverage for mice did not differ from that of voles: electronic supplementary material, S2.1), and/or wood mice may be exposed to fleas from a wider range of sources (e.g. animals living off the trapping grid). This latter hypothesis is potentially supported by the fact that we found a greater proportion of mice than voles to be only caught once (electronic supplementary material, S2.1), implying that mice may be less likely than voles to be permanent residents on a given grid. We also note that we were unable to distinguish *Bartonella* variants in co-infections, which may introduce potential biases in our data, particularly if host-generalist variants are more likely to occur in co-infections than host-specific variants or *vice versa*. Nevertheless, despite the limitations of working with wild systems, and that the use of anti-parasite drug treatments to reduce infections in wildlife can be challenging [22], it is encouraging that we did find evidence that both vole and mouse treatment had an impact on the transmission of VBPs and fleas in this system.

Together, our large-scale perturbation experiment of a natural multihost–multivector–multipathogen system, coupled with genetic characterization of the circulating pathogen strains, revealed that, although transmission dynamics within this system appear complex owing to the potentially high number of host–vector–pathogen combinations, most VBPs show high degrees of specificity for a single host species, and rates of cross-species transmission appear to be low, even for pathogen variants that are found in both host species. These results mirror findings from other VBP systems which show that, although there may be multiple potential host species in a community, VBP transmission is often dominated by few, or only one, host species, although the key host species may vary across different locations (e.g. American robins, blue jays and/or house sparrows for West Nile virus [14,55,56], or the white-footed mouse, shrews or chipmunks for Lyme disease in North America [15,16]). However, different mechanisms may underlie these heterogeneities, owing to varying vector–host feeding preferences (e.g. as has been reported to occur for West Nile Virus; [14,55]), and/or differential pathogen–host compatibilities (e.g. as has been reported for Lyme disease strains; [16,57,58]). Given these complexities, understanding cross-species transmission for VBP systems is highly challenging, but determining the processes driving host–vector–pathogen specificities and heterogeneities will be crucial for effective VBP disease management [16]. We show that fine-scale genetic resolution of the pathogens, and experimental perturbation approaches that block specific transmission pathways, can help uncover the existence and extent of cross-species transmission, and reveal the impact those heterogeneities can have for driving transmission dynamics in such systems.

## Data Availability

Data are available from the Dryad Digital Repository: https://doi.org/10.5061/dryad.zpc866tdx [59], and also in the electronic supplementary material [60]. GenBank accession numbers of all *Bartonella* sequences included in this paper, including those identified for the first time here, are shown in electronic supplementary material, table S3.
